# Association of dietary intake of B vitamins with glaucoma

**DOI:** 10.1038/s41598-024-58526-5

**Published:** 2024-04-12

**Authors:** Jingjing Hou, Yu Wen, Sijia Gao, Zhengxuan Jiang, Liming Tao

**Affiliations:** 1 Department of Ophthalmology, The Second Affiliated Hospital of Anhui Medical University, 678 Furong Road, Hefei, Anhui China; 2 Department of Clinical Medicine, The Second School of Clinical Medicine, Anhui Medical University, 81 Meishan Road, Hefei, Anhui China

**Keywords:** Glaucoma, Vitamin B, Dietary intake, Cross-sectional study, Diseases, Risk factors

## Abstract

This cross-sectional study investigated the association between glaucoma and B vitamin dietary intake. A total of 5025 enrolled individuals participated in self-reported glaucoma questionnaire and 3264 participated in International Society Geographical and Epidemiological Ophthalmology (ISGEO) criteria. In self-reported glaucoma, the risk of having self-reported glaucoma was lower in the third quartile of vitamin B1 intake (odds ratio [odds ratio [OR] 0.63, 95% confidence interval [CI] 0.40–0.97), and P trend (P trend = 0.004) for vitamin B12 was significant; in males, the third quartile of vitamin B1 intake (OR 0.44, 95% CI 0.24–0.83) and the fourth quartile of vitamin B2 intake (OR 0.39, 95% CI 0.17–0.89) were associated with a lower risk. In glaucoma based on ISGEO criteria, the increase of niacin intake (OR 0.94, 95% CI 0.89–0.99) was negatively associated with the odds of self-reported glaucoma. After sex-stratified analysis, the third quartile of vitamin B6 intake (OR 0.21, 95% CI 0.08–0.60) in males were associated with reduced odds of glaucoma. The restricted cubic spline analysis revealed a nonlinear association of vitamin B2 (p for nonlinearity = 0.04) and B9 (p for nonlinearity = 0.024) intake with glaucoma diagnosed by ISGEO criteria in females.

## Introduction

The global prevalence of glaucoma, a serious irreversible eye-blinding disease, is about 3.5% in the population over 40 years of age. Primary open-angle glaucoma (POAG), a common type of glaucoma in European populations, has a global prevalence of about 3.1%^1,2^. With the aging of the population, the prevalence of glaucoma will increase significantly in the future, resulting in social, medical, and economic burdens^3^. However, the mechanisms underlying retinal ganglion cell (RGC) apoptosis in glaucoma remain unclear. Increased intraocular pressure (IOP) is a leading factor of glaucomatous visual impairment and the effective intervention point^4,5^.

Currently, most treatments are based on reducing IOP, which reduces damage to RGCs^6^. Oxidative stress is one of the mechanisms of RGC damage. Free radical species can interfere with the tricarboxylic acid cycle and mitochondrial metabolic pathways, leading to RGC death^7,8^. However, specific nutrients may act as antioxidants to improve and protect nerve cell function^9–11^. Niacin (vitamin B3), as a precursor of total nicotinamide adenine dinucleotide (NAD), can slow mitochondrial dysfunction and lessen optic nerve damage in glaucoma^12,13^. Serum homocysteine levels are elevated in patients with POAG and pseudoexfoliation glaucoma (PEXG)^14,15^. Serum vitamin B6, B9, and B12 levels are closely related to serum homocysteine concentrations, which can induce oxidative stress to accelerate the degeneration and apoptosis of RGCs^16^. Kang et al.^17^ explored whether increased intake of vitamins B6 and B12 and folic acid (vitamin B9) could reduce the risk of exfoliative glaucoma (EG) by reducing the oxidative stress effect of homocysteine, and reported that a higher total intake of folic acid was associated with a reduced EG risk. Oxidative stress markers are significantly increased in the aqueous humor in different glaucoma types^18^. B vitamins, as antioxidants, can protect the function of nerve cells by reducing oxidative stress in RGCs.

In this study, we investigated the potential impact of the daily intake of vitamins B1, B2, B6, B12, niacin, and folic acid on glaucoma using the National Health and Nutrition Examination Survey (NHANES), a large population-based study in the United States of America (US) with the aim to understand the association between vitamin B intake and glaucoma prevalence.

## Materials and methods

### Sample population

We used publicly available data from the 2005–2006 and 2007–2008 NHANES, which entailed conducting cross-sectional interviews and examinations of approximately 10,000 US non-institutionalized civilians in every cycle. The NHANES uses a stratified multi-stage sampling design and weighting scheme to accurately determine disease prevalence in the US population. All NHANES protocols were approved by the National Center for Health Statistics Ethics Review Board of the Centers for Disease Control (Protocol #2005-06, Continuation of Protocol #2005-06), and all survey participants provided written informed consent. The study followed the tenets of the Declaration of Helsinki.

We analyzed the public data from 2005 to 2008 in NHANES from 7081 participants aged over 40 years. The self-reported glaucoma exclusion criteria were results of at least one dietary interview for lacking B vitamins, no response to self-glaucoma interview and taking nutritional supplements; this led to the exclusion of 63931 and 1386 participants, respectively. Thus, 5025 eligible participants were included in the self-rated glaucoma primary outcome analysis. The secondary outcome was based on the International Society Geographical and Epidemiological Ophthalmology (ISGEO) criteria for glaucoma diagnosis based on retinal imaging and frequency doubling technology (FDT). Additional exclusion criteria based on self-rated glaucoma were incomplete, insufficient, or unreliable FDT analysis, and insufficient or unreliable retinal imaging. Accordingly, we excluded 1,560 and 201 participants, respectively. We included 3264 eligible participants in the secondary outcome analysis.

### Assessment of glaucoma

The primary outcome was the severity of self-reported glaucoma. Responses were obtained using a visual questionnaire. When asked, “Have you ever been told by an ophthalmologist that you had glaucoma, sometimes called high pressure in eyes?”. participants who refused to answer or stated they did not know the answer, were excluded; 331 answered “yes.”

The secondary outcome variable was glaucoma diagnosis using the ISGEO criteria based on retinal imaging and FDT. Retinal imaging refers to technicians using a Canon Non-Mydriatic Retinal Camera CR6-45 NM to capture 45°-non-mydriatic digital retina images in a dark room. The participants were required to focus on the target for proper positioning. Two digital images per eye are captured at the same time, with the first image centered on the macula and the second image centered on the optic nerve. The images are first shipped to the University of Wisconsin for scoring. In 2012, ophthalmologists at Johns Hopkins University re-read retinal images with a cup-disc ratio (CDR) of 0.6 or higher to look for other indicators indicating the presence of glaucoma. The N-30-5 FDT screening protocol was designed to test visual field loss owing to eye diseases, especially glaucoma, using the Humphrey Matrix Visual Field Instrument. Each participant was tested twice, including 19 visual field locations in each eye. Each visual field location was tested until participants responded. A positive FDT result was obtained when at least two positions fell below a 1% threshold level in the first and second tests, and at least one failure position was the same in both tests (2-2-1 algorithm). Incomplete or unreliable test results were excluded from the study.

Combined with the results of the NHANES examination, we adopted classifications 1 and 2 of the ISGEO diagnostic criteria^19,20^: (1) positive FDT results in at least one eye with a cup-disc ratio (CDR) ≥ 97.5th percentile in the same eye or with a CDR asymmetry ≥ 97.5th percentile for the NHANES participants with normal visual function (normal visual field); and (2) CDR ≥ 99.5th percentile in either eye or CDR asymmetry between eyes ≥ 99.5th percentile for the NHANES participants with normal visual function (normal visual field), regardless of the FDT results. We diagnosed 108 participants with glaucoma based on the ISGEO criteria.

### Assessment of dietary B vitamins

The main predictors were the daily intake of vitamins B1, B2, B6, B12, niacin, and folic acid derived from the NHANES Dietary Interview-Total Nutrient Intakes. This information was used to estimate the type and amount of food and drink consumed 24-h before the interview (midnight to midnight) and to calculate the intake of energy, nutrients, and other food components. All NHANES participants underwent two 24-h dietary recall interviews. The first dietary recall interview was conducted at the mobile examination center, and the second was conducted via telephone 3–10 days later. We used the average of the two 24-h dietary intakes as the final dietary intake data, and participant lacking the second 24-h dietary intake data used the first 24-h dietary intake as the final data.

### Assessment of covariates

We included age, sex, race, educational level, smoke, total energy intake, caffeine intake, diabetes, and cataract surgery as covariates^12,21,22^. Vitamins B2 and B6 are closely related because the interconversion of some vitamin B6 species requires the vitamin B2 forms flavin mononucleotide (FMN) and flavin dinucleotide (FAD) as cofactors^23^. At the same time, vitamins B2 and B6 are necessary cofactors for converting tryptophan into niacin^24^. Folic acid and vitamin B12 coordinate and play an important role in the treatment of megaloblastic anemia (MA) and in improving the metabolism of the central nervous system^25^. Considering the interaction between B vitamins, additional covariates of different B vitamins are added: vitamin B2: add vitamin B6; niacin: add vitamin B2 and vitamin B6; vitamin B6: add vitamin B2; folic acid: add vitamin B12; vitamin B12: add folic acid.

### Data analyses

Descriptive statistics were used to assess the baseline characteristics of the study population. Age, total energy intake, caffeine intake, and B vitamins intake were analyzed as continuous variables, whereas sex, race, educational level, household income, smoking status, diabetes status, and cataract surgery as categorical variables. The distributions of these variables between participants with and without self-reported glaucoma were compared using design-adjusted Rao–Scott (Pearson-type) χ^2^ and Wald tests for categorical and continuous variables, respectively. All data were weighted by NHANES to produce weighted estimates representing the US population.

Logistic regression modeling was used to examine the association between daily dietary vitamin B consumption and glaucoma with B vitamins as continuous and categorical variable in quartiles. The reference of quartiles was determined based on recommended daily allowances (RDAs), proposed by the National Institutes of Health, which was sufficient to meet the nutritional needs of 97–98% healthy individuals and was commonly used to plan for a nutritionally adequate diet for individuals in Supplement Table S1^26^. Supplemental Table S1 provides the overall RDAs based on the male-to-female ratio of self-reported participants to facilitate the determination of the overall reference value. Trend analysis was performed by modeling the median within the quartiles as a continuous covariate. Crude model is an unadjusted model. Model I was adjusted for socio-demographic characteristics and some factors where p values were less than 0.05, including age, sex, race, and education level. Model II was adjusted comprehensively, including age, sex, race, educational level, smoke, total energy intake, caffeine intake, diabetes, cataract surgery, and interacted B vitamins. Additionally, we performed multivariable-adjusted restricted cubic splines (RCS) with 3 knots at the 10th, 50th, and 90th percentiles to examine potential non-linear associations between the dietary intake of B vitamins and glaucoma prevalence after controlling for all confounders. The test level was α = 0.05, and P < 0.05 was considered statistically significant. The analyses were performed using Stata 16.1 (Stata Corp LP, College Station, TX, USA) and R software version 4.2.2.

## Results

**Figure 1:**
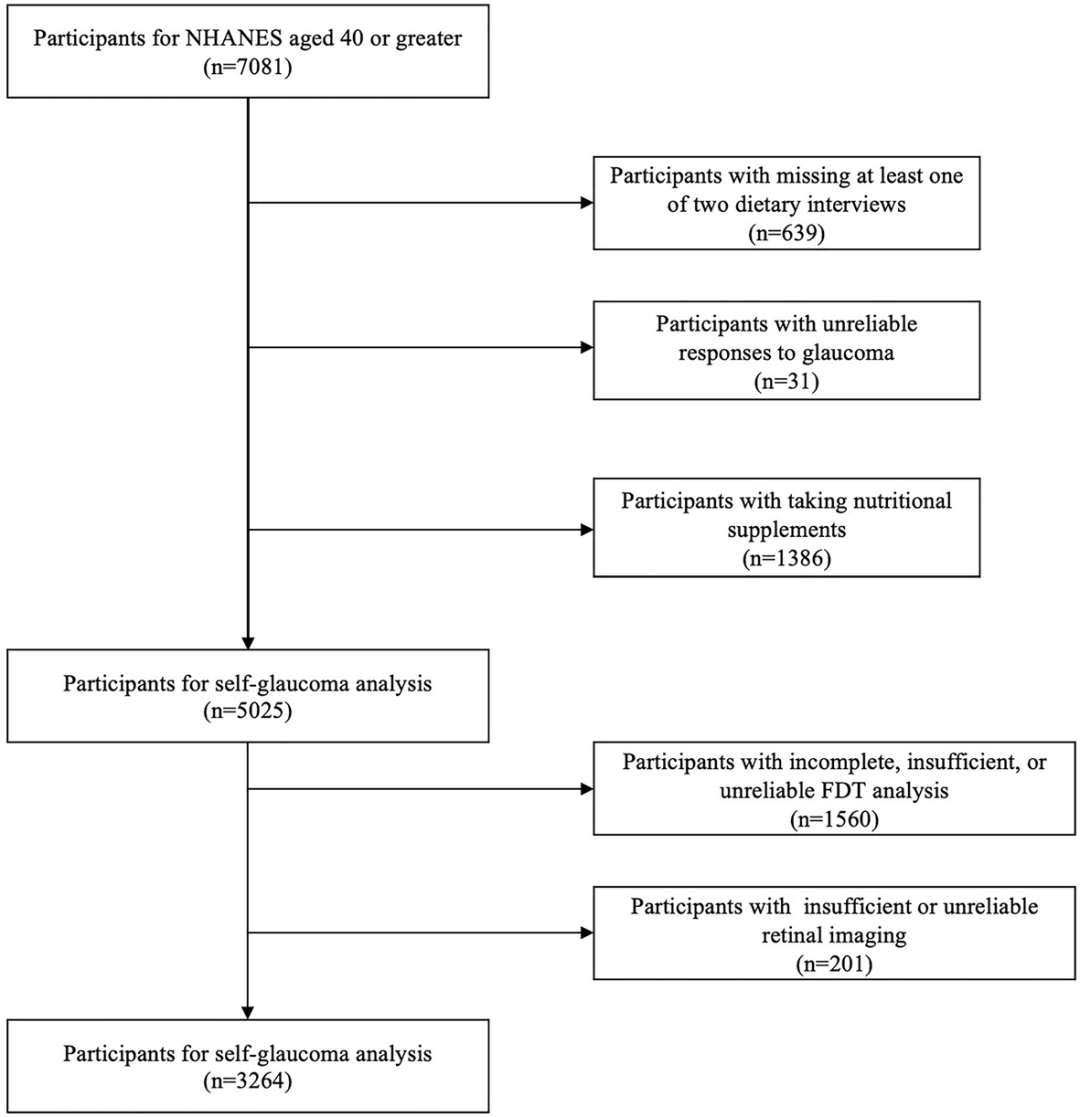
Flowchart of the study population.

The NHANES database included 5025 participants aged over 40 years, with reliable glaucoma questionnaire responses and B vitamin dietary interview results from 2005 to 2008. The female individuals comprised 49.19% (2472/5025) of the population. A total of 331 (6.59%) participants had self-reported glaucoma. The database included 3264 patients with reliable retinal imaging, FDT visual field results, and B vitamin dietary interviews to assess glaucoma characteristics based on the ISGEO criteria, including 108 participants with glaucoma (3.31%). The flowchart of participants selection is presented in Fig. 1. Table 1 presents the demographic characteristics of participants with and without self-reported glaucoma. Compared with participants without glaucoma, participants with self-reported glaucoma were older (P < 0.001), had a more distinct race-ethnic distribution (P = 0.004), lower levels of education (P = 0.006), higher rates of smoking (P < 0.001), diabetes (P < 0.001), cataract surgery (P < 0.001), higher intake levels of daily total energy (P < 0.001), caffeine intake (P = 0.008), and lower intake levels of B vitamins (all P < 0.01).

**Table 1:** Demographic and characteristics of participants with or without self-reported glaucoma.

Characteristic	Self-reported glaucoma		P value*
	Yes (n = 331)	No (n = 4694)	
Age (mean ± SD, years)	66.21 ± 12.35	55.84 ± 11.91	< 0.001**
Sex%(SE)			0.761
Male	49.49 (3.53)	48.51 (0.93)	
Female	50.51 (3.53)	51.49 (0.93)	
Race/ethnicity%(SE)			0.004
Mexican American	4.63 (0.84)	6.21 (0.25)	
Other Hispanic	2.43 (0.54)	3.36 (0.27)	
Non-hispanic white	69.80 (2.79)	74.59 (0.67)	
Non-hispanic black	18.59 (2.01)	10.77 (0.38)	
Other race	4.56 (1.81)	5.06 (0.44)	
Education level%(SE)			0.006
Less than 9th grade	12.89 (1.92)	8.08 (0.38)	
9–11th grade	12.86 (2.08)	12.00 (0.53)	
High school grade	26.36 (3.29)	26.70 (0.83)	
Some college	28.21 (3.32)	28.01 (0.85)	
College graduate or above	16.69 (2.78)	25.22 (0.86)	
Annual household income%(SE)			0.198
< $35,000	52.90 (3.57)	48.73 (0.93)	
≥ $35,000	47.10 (3.57)	51.27 (0.93)	
Smoking status%(SE)			< 0.001
Never	46.45 (3.52)	47.78 (0.93)	
Former	40.70 (3.50)	29.21 (0.84)	
Current	12.86 (2.38)	23.02 (0.79)	
Diabetes%(SE)			< 0.001
Yes	23.74 (2.74)	11.11 (0.52)	
No	76.26 (2.74)	88.89 (0.52)	
Cataract surgery%(SE)			< 0.001
Yes	31.49 (3.17)	8.61 (0.44)	
No	68.51 (3.17)	91.39 (0.44)	
Alcohol intake%(SE)			0.201
Yes	63.64 (3.28)	68.79 (0.84)	
No	31.14 (3.16)	26.12 (0.79)	
Not recorded	5.23 (1.26)	5.09 (0.40)	
Vitamin B1 (mean ± SD, mg)	1.41 ± 0.64	1.61 ± 0.80	< 0.001**
Vitamin B2 (mean ± SD, mg)	1.94 ± 0.82	2.23 ± 1.08	< 0.001**
Niacin (mean ± SD, mg)	20.66 ± 7.94	24.47 ± 11.67	< 0.001**
Vitamin B6 (mean ± SD, mg)	1.72 ± 0.83	1.97 ± 1.05	< 0.001**
Folic acid (mean ± SD, mcg)	344.14 ± 149.47	397.72 ± 202.47	< 0.001**
Vitamin B12 (mean ± SD, mcg)	5.35 ± 4.82	6.54 ± 7.16	0.009**
Daily total energy (mean ± SD, kcal)	1739.39 ± 617.78	2041.77 ± 825.05	< 0.001**
Caffeine intake (mean ± SD, mg)	169.54 ± 228.13	207.61 ± 222.24	0.008**

*SE* standard error

*Design-adjusted Rao-Scott Chi-squared test, **Adjusted Wald test.

The quartiles of the daily intake of B vitamins are presented in Table 2.

**Table 2:** Quartile categories of vitamin B intake.

Nutrient	Q1	Q2	Q3	Q4
Overall				
Vitamin B1 (mg/day)	< 1.011	≥ 1.011	≥ 1.386	≥ 1.855
Vitamin B2 (mg/day)	< 1.339	≥ 1.339	≥ 1.877	≥ 2.52
Niacin (mg/day)	< 15.014	≥ 15.014	≥ 20.729	≥ 28.433
Vitamin B6 (mg/day)	< 1.179	≥ 1.179	≥ 1.658	≥ 2.285
Folic acid (mcg/day)	< 241.5	≥ 241.5	≥ 336	≥ 462.75
Vitamin B12 (mcg/day)	< 2.515	≥ 2.515	≥ 4.31	≥ 7.308
Male				
Vitamin B1 (mg/day)	< 1.159	≥ 1.159	≥ 1.601	≥ 2.097
Vitamin B2 (mg/day)	< 1.509	≥ 1.509	≥ 2.129	≥ 2.834
Niacin (mg/day)	< 17.615	≥ 17.615	≥ 24.343	≥ 32.605
Vitamin B6 (mg/day)	< 1.369	≥ 1.369	≥ 1.914	≥ 2.669
Folic acid (mcg/day)	< 270	≥ 270	≥ 375	≥ 514
Vitamin B12 (mcg/day)	< 2.945	≥ 2.945	≥ 4.983	≥ 8.463
Female				
Vitamin B1 (mg/day)	< 0.902	≥ 0.902	≥ 1.208	≥ 1.58
Vitamin B2 (mg/day)	< 1.223	≥ 1.223	≥ 1.664	≥ 2.173
Niacin (mg/day)	< 13.286	≥ 13.286	≥ 17.709	≥ 23.462
Vitamin B6 (mg/day)	< 1.048	≥ 1.048	≥ 1.45	≥ 1.961
Folic acid (mcg/day)	< 219.5	≥ 219.5	≥ 300	≥ 401
Vitamin B12 (mcg/day)	< 2.25	≥ 2.25	≥ 3.665	≥ 6.195

We examined the association between daily B vitamins dietary intake and self-reported glaucoma prevalence (Table 3). Meanwhile, we conducted a further sex-stratification analysis (male: Table 4; female: Supplement Table S2). Overall analysis showed that vitamin B1 consumption at the third quartile (crude model: OR 0.55, 95% CI 0.36–0.82 P = 0.004; model I: OR 0.58, 95% CI 0.38–0.89, P = 0 0.013; model II: OR 0.63, 95% CI 0.40–0.97, P = 0.036) had significantly decreased odds of self-reported glaucoma compared with that at the second quartile in all models. Additionally, the odds of self-reported glaucoma showed a decreasing trend with higher quartiles of vitamin B12 intake (crude model: P trend < 0.001; model I: P trend < 0.001; model II: P trend = 0.004).

**Table 3:** Association between daily dietary intake of B vitamins and self-reported glaucoma prevalence.

	Crude model OR (95% CI)	P value	Model I OR (95% CI)	P value	Model II OR (95% CI)	P value
Vitamin B1						
Continuous	0.67 (0.53, 0.84)	< 0.001	0.73 (0.56, 0.97)	0.029	0.84 (0.61, 1.14)	0.26
Q1	1.03 (0.70, 1.51)	0.872	1.10 (0.74, 1.65)	0.64	1.09 (0.72, 1.66)	0.69
Q2	Ref		Ref		Ref	
Q3	0.55 (0.36, 0.82)	0.004	0.58 (0.38, 0.89)	0.013	0.63 (0.40, 0.97)	0.036
Q4	0.53 (0.35, 0.80)	0.002	0.63 (0.40, 1.01)	0.056	0.74 (0.45, 1.23)	0.244
p trend		< 0.001		0.011		0.122
Vitamin B2						
Continuous	0.73 (0.63, 0.85)	< 0.001	0.80 (0.67, 0.95)	0.012	0.85 (0.69, 1.06)	0.156
Q1	Ref		Ref		Ref	
Q2	0.94 (0.64, 1.37)	0.746	1.02 (0.68, 1.53)	0.907	1.07 (0.71, 1.63)	0.737
Q3	0.68 (0.45, 1.02)	0.065	0.73 (0.47, 1.14)	0.165	0.81 (0.51, 1.28)	0.369
Q4	0.48 (0.31, 0.72)	< 0.001	0.59 (0.37, 0.96)	0.033	0.72 (0.39, 1.32)	0.289
p trend		< 0.001		0.009		0.118
Niacin						
Continuous	0.96 (0.95, 0.98)	< 0.001	0.98 (0.96, 0.99)	0.007	0.99 (0.97, 1.02)	0.521
Q1	0.99 (0.68, 1.44)	0.962	0.87 (0.59, 1.29)	0.495	0.71 (0.47, 1.08)	0.114
Q2	Ref		Ref		Ref	
Q3	0.66 (0.44, 0.98)	0.038	0.73 (0.48, 1.11)	0.142	0.85 (0.54, 1.32)	0.461
Q4	0.48 (0.31, 0.74)	0.001	0.65 (0.39, 1.07)	0.088	1.04 (0.56, 1.92)	0.899
p trend		< 0.001		0.051		0.505
Vitamin B6						
Continuous	0.76 (0.65, 0.89)	0.001	0.81 (0.67, 0.96)	0.018	0.96 (0.79, 1.16)	0.665
Q1	1.17 (0.80, 1.72)	0.42	1.10 (0.74, 1.65)	0.635	1.03 (0.68, 1.58)	0.884
Q2	Ref		Ref		Ref	
Q3	0.76 (0.50, 1.16)	0.204	0.77 (0.50, 1.20)	0.25	0.87 (0.56, 1.34)	0.518
Q4	0.63 (0.42, 0.94)	0.024	0.68 (0.44, 1.05)	0.081	0.93 (0.59, 1.47)	0.756
p trend		0.005		0.038		0.63
Folic acid						
Continuous	1.00 (1.00, 1.00)	< 0.001	1.00 (1.00, 1.00)	0.002	1.00 (1.00, 1.00)	0.228
Q1	1.45 (0.97, 2.15)	0.067	1.37 (0.90, 2.08)	0.145	1.21 (0.75, 1.97)	0.431
Q2	1.27 (0.85, 1.90)	0.251	1.16 (0.76, 1.77)	0.481	1.08 (0.70, 1.67)	0.714
Q3	Ref		Ref		Ref	
Q4	0.62 (0.40, 0.96)	0.032	0.65 (0.42, 1.03)	0.065	0.78 (0.50, 1.21)	0.264
p trend		0.001		0.01		0.155
Vitamin B12						
Continuous	0.96 (0.93, 0.99)	0.025	0.96 (0.93, 1.00)	0.036	0.98 (0.95, 1.01)	0.238
Q1	Ref		Ref		Ref	
Q2	1.00 (0.67, 1.51)	0.987	1.07 (0.70, 1.63)	0.753	1.14 (0.74, 1.75)	0.559
Q3	0.89 (0.60, 1.32)	0.569	0.88 (0.58, 1.33)	0.532	1.00 (0.65, 1.54)	0.994
Q4	0.55 (0.36, 0.84)	0.006	0.54 (0.34, 0.85)	0.008	0.70 (0.42, 1.16)	0.162
p trend		< 0.001		< 0.001		0.004

Model I adjusted for age, sex, race and educational level. Model II adjusted for age, sex, race, educational level, smoking, diabetes, cataract surgery, daily total energy, caffeine intake and interacted vitamin b.

**Table 4:** Association between daily dietary intake of B vitamins and self-reported glaucoma prevalence in males.

	Crude model OR (95% CI)	P value	Model I OR (95% CI)	P value	Model II OR (95% CI)	P value
Vitamin B1						
Continuous	0.47 (0.32, 0.69)	< 0.001	0.55 (0.36, 0.83)	0.005	0.68 (0.43, 1.08)	0.101
Q1	0.99 (0.58, 1.67)	0.961	1.09 (0.62, 1.91)	0.773	1.00 (0.56, 1.78)	0.992
Q2	Ref		Ref		Ref	
Q3	0.33 (0.18, 0.61)	< 0.001	0.39 (0.21, 0.71)	0.002	0.44 (0.24, 0.83)	0.012
Q4	0.30 (0.16, 0.57)	< 0.001	0.42 (0.22, 0.82)	0.011	0.56 (0.27, 1.13)	0.105
p trend		< 0.001		< 0.001		0.023
Vitamin B2						
Continuous	0.61 (0.48, 0.78)	< 0.001	0.66 (0.51, 0.86)	0.002	0.72 (0.53, 0.97)	0.029
Q1	Ref		Ref		Ref	
Q2	0.72 (0.42, 1.25)	0.245	0.67 (0.37, 1.20)	0.177	0.75 (0.41, 1.41)	0.376
Q3	0.65 (0.37, 1.17)	0.151	0.68 (0.36, 1.27)	0.223	0.85 (0.41, 1.74)	0.655
Q4	0.25 (0.13, 0.47)	< 0.001	0.29 (0.14, 0.59)	0.001	0.39 (0.17, 0.89)	0.025
p trend		< 0.001		0.002		0.051
Niacin						
Continuous	0.94 (0.92, 0.96)	< 0.001	0.96 (0.94, 0.98)	< 0.001	0.98 (0.94, 1.02)	0.275
Q1	Ref		Ref		Ref	
Q 2	0.65 (0.38, 1.12)	0.119	0.75 (0.42, 1.36)	0.35	1.03 (0.54, 1.96)	0.94
Q3	0.35 (0.19, 0.62)	< 0.001	0.48 (0.25, 0.92)	0.026	0.89 (0.42, 1.87)	0.753
Q4	0.28 (0.15, 0.53)	< 0.001	0.47 (0.23, 0.96)	0.037	1.36 (0.47, 3.90)	0.569
p trend		< 0.001		0.024		0.617
Vitamin B6						
Continuous	0.62 (0.48, 0.79)	< 0.001	0.69 (0.53, 0.89)	0.004	0.96 (0.75, 1.23)	0.733
Q1	1.29 (0.75, 2.21)	0.354	1.11 (0.64, 1.94)	0.706	0.84 (0.48, 1.47)	0.537
Q2	Ref		Ref		Ref	
Q3	0.48 (0.26, 0.89)	0.019	0.48 (0.26, 0.91)	0.024	0.55 (0.29, 1.06)	0.075
Q4	0.44 (0.24, 0.81)	0.008	0.49 (0.26, 0.93)	0.028	0.89 (0.46, 1.74)	0.737
p trend		< 0.001		0.005		0.656
Folic acid						
Continuous	1.00 (1.00, 1.00)	< 0.001	1.00 (1.00, 1.00)	0.001	1.00 (1.00, 1.00)	0.296
Q1	1.78 (1.01, 3.12)	0.045	1.57 (0.85, 2.88)	0.147	1.05 (0.51, 2.16)	0.904
Q2	0.97 (0.53, 1.75)	0.91	0.85 (0.47, 1.54)	0.589	0.64 (0.34, 1.21)	0.166
Q3	Ref		Ref		Ref	
Q4	0.57 (0.30, 1.09)	0.09	0.63 (0.33, 1.19)	0.155	0.80 (0.43, 1.47)	0.467
p trend		0.007		0.027		0.678
Vitamin B12						
Continuous	0.93 (0.87, 1.00)	0.036	0.94 (0.88, 1.00)	0.061	0.97 (0.92, 1.02)	0.296
Q1	Ref		Ref		Ref	
Q2	1.01 (0.58, 1.76)	0.965	1.09 (0.62, 1.91)	0.771	1.22 (0.68, 2.18)	0.508
Q3	0.58 (0.32, 1.05)	0.072	0.59 (0.32, 1.10)	0.094	0.76 (0.41, 1.43)	0.399
Q4	0.31 (0.15, 0.62)	0.001	0.33 (0.16, 0.69)	0.002	0.48 (0.22, 1.06)	0.069
p trend		0.002		0.002		0.093

Model I adjusted for age, race and educational level. Model II adjusted for age, race, educational level, smoking, diabetes, cataract surgery, daily total energy, caffeine intake and interacted vitamin b.

In males, there was a significant negative association with the odds of self-reported glaucoma observed in the third quartile (crude model: OR 0.33, 95% CI 0.18–0.61 P < 0.001; model I: OR 0.39, 95% CI 0.21–0.71, P = 0 0.002; model II: OR 0.44, 95% CI 0.24–0.83, P = 0.012) quartile of vitamin B1 compared with that in the second quartile in the fully adjusted model. Trend analysis showed the adjusted odds of glaucoma were reduced with higher vitamin B1 consumption (crude model: P trend < 0.001; model I: P trend < 0.001; model II: P trend = 0.023).We also found vitamin B2 intake as a continuous variable, with a 28% reduction in glaucoma risk for every 1 mg increase (crude model: OR 0.61, 95% CI 0.48–0.78 P < 0.001; model I: OR 0.66, 95% CI 0.51–0.86, P = 0 0.002; model II: OR 0.72, 95% CI 0.53–0.97, P = 0.029). Furthermore, high quartile vitamin B2 intake was significantly associated with the risk of glaucoma (crude model: OR 0.25, 95% CI 0.13–0.47 P < 0.001; model I: OR 0.29, 95% CI 0.14–0.59, P = 0.001; model II: OR 0.39, 95% CI 0.17–0.89, P = 0.025). No association was observed between the daily consumption of other B vitamins and the odds of glaucoma prevalence. Moreover, we did not find an association between intake B vitamins and self- reported glaucoma in females.

We examined the association between the daily dietary intake of B vitamins and glaucoma diagnosis using the ISGEO criteria (Table 5). Additionally, we conducted a sex-stratification analysis (male: Table 6; female: Supplement Table S3). Overall analysis suggested that niacin analyzed as a continuous variable was associated with lower odds of glaucoma in all models (crude model: OR 0.97, 95% CI 0.94–0.99, P = 0.011; model I: OR 0.97, 95% CI 0.95–1.00, P = 0.048; model II: OR 0.94, 95% CI 0.89–0.99, P = 0.031).

**Table 5:** Association between daily B vitamins and glaucoma diagnosed by ISGEO criteria.

	Crude model OR (95% CI)	P value	Model I OR (95% CI)	P value	Model II OR (95% CI)	P value
Vitamin B1						
Continuous	0.86 (0.59, 1.25)	0.425	0.91 (0.59, 1.39)	0.665	0.94 (0.55, 1.60)	0.822
Q1	0.48 (0.24, 0.98)	0.044	0.50 (0.24, 1.05)	0.066	0.50 (0.24, 1.05)	0.069
Q2	Ref		Ref		Ref	
Q3	0.48 (0.24, 0.98)	0.043	0.51 (0.25, 1.04)	0.064	0.51 (0.24, 1.08)	0.074
Q4	0.46 (0.23, 0.93)	0.03	0.50 (0.24, 1.05)	0.068	0.50 (0.23, 1.08)	0.077
p trend		0.189		0.198		0.419
Vitamin B2						
Continuous	0.83 (0.63, 1.09)	0.183	0.89 (0.67, 1.18)	0.417	1.07 (0.74, 1.53)	0.717
Q1	Ref		Ref		Ref	
Q2	0.86 (0.43, 1.75)	0.685	0.87 (0.41, 1.84)	0.711	0.85 (0.39, 1.84)	0.676
Q3	0.81 (0.38, 1.70)	0.571	0.84 (0.39, 1.81)	0.652	0.92 (0.39, 2.20)	0.857
Q4	0.55 (0.25, 1.20)	0.133	0.61 (0.27, 1.40)	0.243	0.73 (0.25, 2.17)	0.571
p trend		0.161		0.168		0.665
Niacin						
Continuous	0.97 (0.94, 0.99)	0.011	0.97 (0.95, 1.00)	0.048	0.94 (0.89, 0.99)	0.031
Q1	Ref		Ref		Ref	
Q2	0.82 (0.43, 1.59)	0.561	0.79 (0.40, 1.53)	0.483	0.86 (0.42, 1.76)	0.688
Q3	0.53 (0.26, 1.10)	0.089	0.52 (0.25, 1.06)	0.074	0.48 (0.22, 1.02)	0.057
Q4	0.45 (0.21, 0.95)	0.036	0.49 (0.22, 1.06)	0.07	0.36 (0.11, 1.21)	0.099
p trend		0.034		0.07		0.165
Vitamin B6						
Continuous	0.92 (0.69, 1.22)	0.57	0.94 (0.67, 1.32)	0.73	0.91 (0.61, 1.37)	0.664
Q1	0.79 (0.40, 1.58)	0.508	0.81 (0.40, 1.64)	0.56	0.84 (0.41, 1.72)	0.63
Q2	Ref		Ref		Ref	
Q3	0.50 (0.23, 1.11)	0.088	0.51 (0.22, 1.16)	0.106	0.49 (0.21, 1.14)	0.097
Q4	0.71 (0.35, 1.43)	0.331	0.73 (0.34, 1.58)	0.429	0.69 (0.28, 1.69)	0.417
p trend		0.364		0.344		0.374
Folic acid						
Continuous	1.00 (1.00, 1.00)	0.344	1.00 (1.00, 1.00)	0.431	1.00 (1.00, 1.00)	0.408
Q1	0.87 (0.41, 1.83)	0.709	0.87 (0.40, 1.85)	0.704	0.81 (0.35, 1.92)	0.637
Q2	1.14 (0.56, 2.33)	0.709	1.08 (0.53, 2.21)	0.825	1.09 (0.53, 2.25)	0.814
Q3	Ref		Ref		Ref	
Q4	0.90 (0.43, 1.87)	0.78	0.92 (0.42, 1.98)	0.823	0.98 (0.44, 2.15)	0.955
p trend		0.841		0.751		0.834
Vitamin B12						
Continuous	1.00 (0.97, 1.03)	0.93	1.00 (0.97, 1.03)	0.99	1.00 (0.98, 1.03)	0.643
Q1	Ref		Ref		Ref	
Q2	0.97 (0.46, 2.04)	0.931	1.05 (0.48, 2.29)	0.906	1.04 (0.45, 2.39)	0.922
Q3	1.03 (0.49, 2.16)	0.929	1.07 (0.49, 2.33)	0.87	1.17 (0.47, 2.87)	0.74
Q4	0.99 (0.50, 1.99)	0.987	1.02 (0.47, 2.19)	0.961	1.24 (0.45, 3.43)	0.679
p trend		0.928		0.782		0.581

Model I adjusted for age, sex, race and educational level. Model II adjusted for age, sex, race, educational level, smoking, diabetes, cataract surgery, daily total energy, caffeine intake and interacted vitamin b.

**Table 6:** Association between daily B vitamins and glaucoma diagnosed by ISGEO criteria in males.

	Crude model OR (95% CI)	P value	Model I OR (95% CI)	P value	Model II OR (95% CI)	P value
Vitamin B1						
Continuous	0.71 (0.45, 1.13)	0.145	0.81 (0.49, 1.32)	0.399	0.68 (0.39, 1.20)	0.182
Q1	1.35 (0.54, 3.39)	0.525	1.42 (0.54, 3.74)	0.475	1.51 (0.55, 4.14)	0.419
Q2	Ref		Ref		Ref	
Q3	0.60 (0.19, 1.89)	0.386	0.67 (0.22, 2.11)	0.497	0.58 (0.19, 1.81)	0.35
Q4	0.75 (0.29, 1.92)	0.547	1.00 (0.35, 2.80)	0.996	0.84 (0.29, 2.45)	0.75
p trend		0.11		0.247		0.193
Vitamin B2						
Continuous	0.88 (0.65, 1.20)	0.433	0.99 (0.72, 1.35)	0.948	1.23 (0.82, 1.83)	0.318
Q1	Ref		Ref		Ref	
Q2	1.29 (0.53, 3.10)	0.574	1.23 (0.53, 2.90)	0.628	1.27 (0.55, 2.95)	0.58
Q3	0.83 (0.30, 2.33)	0.727	0.85 (0.31, 2.33)	0.757	0.94 (0.26, 3.41)	0.924
Q4	0.72 (0.30, 1.72)	0.463	0.91 (0.37, 2.21)	0.828	1.13 (0.39, 3.30)	0.822
p trend		0.267		0.408		0.873
Niacin						
Continuous	0.96 (0.93, 0.99)	0.016	0.98 (0.94, 1.01)	0.183	0.93 (0.86, 1.01)	0.085
Q1	Ref		Ref		Ref	
Q2	0.57 (0.21, 1.53)	0.268	0.62 (0.24, 1.58)	0.317	0.53 (0.19, 1.51)	0.235
Q3	0.66 (0.28, 1.58)	0.353	0.81 (0.32, 2.00)	0.64	0.62 (0.21, 1.89)	0.405
Q4	0.35 (0.13, 0.98)	0.047	0.55 (0.20, 1.49)	0.237	0.36 (0.04, 2.94)	0.337
p trend		0.05		0.295		0.457
Vitamin B6						
Continuous	0.89 (0.58, 1.36)	0.593	0.97 (0.62, 1.52)	0.893	0.84 (0.48, 1.47)	0.534
Q1	1.17 (0.45, 3.04)	0.748	1.11 (0.43, 2.86)	0.83	1.27 (0.45, 3.55)	0.651
Q2	Ref		Ref		Ref	
Q3	0.27 (0.10, 0.72)	0.009	0.26 (0.09, 0.76)	0.014	0.21 (0.08, 0.60)	0.004
Q4	0.69 (0.26, 1.79)	0.44	0.76 (0.27, 2.18)	0.611	0.47 (0.14, 1.59)	0.225
p trend		0.111		0.216		0.036
Folic acid						
Continuous	1.00 (1.00, 1.00)	0.254	1.00 (1.00, 1.00)	0.445	1.00 (1.00, 1.00)	0.324
Q1	0.88 (0.31, 2.47)	0.81	0.80 (0.28, 2.27)	0.68	0.84 (0.25, 2.81)	0.78
Q2	1.13 (0.44, 2.93)	0.796	1.04 (0.41, 2.63)	0.929	1.02 (0.40, 2.56)	0.969
Q3	Ref		Ref		Ref	
Q4	0.68 (0.24, 1.94)	0.472	0.70 (0.23, 2.15)	0.537	0.66 (0.19, 2.26)	0.512
p trend		0.264		0.288		0.249
Vitamin B12						
Continuous	0.98 (0.93, 1.04)	0.517	0.99 (0.94, 1.04)	0.588	0.99 (0.95, 1.04)	0.826
Q1	Ref		Ref		Ref	
Q2	0.91 (0.34, 2.40)	0.848	0.94 (0.35, 2.49)	0.897	0.86 (0.30, 2.51)	0.787
Q3	0.49 (0.17, 1.41)	0.183	0.50 (0.17, 1.52)	0.224	0.46 (0.12, 1.72)	0.247
Q4	0.62 (0.23, 1.65)	0.337	0.65 (0.24, 1.80)	0.409	0.65 (0.17, 2.54)	0.534
p trend		0.227		0.213		0.282

Model I adjusted for age, race and educational level. Model II adjusted for age, race, educational level, smoking, diabetes, cataract surgery, daily total energy, caffeine intake and interacted vitamin b.

In males, vitamin B6 intake at the third quartile (crude model: OR 0.27, 95% CI 0.10–0.72, P = 0.009; model I: OR 0.26 95% CI 0.09–0.76, P = 0.014; model II: OR 0.21, 95% CI 0.08–0.60, P = 0.031) quartile had significantly decreased odds of glaucoma compared with that at the second quartile in all models. We observed no association between other B vitamins and the odds of glaucoma. Sex-stratified analysis indicated no significant association between vitamin B and the odds of glaucoma. After trend analysis, we discovered a significant P trend value for vitamin B6 in model II (model II: P trend = 0.036). Sex-stratified analysis indicated no significant association between vitamin B and the odds of glaucoma in females.

**Figure 2:**
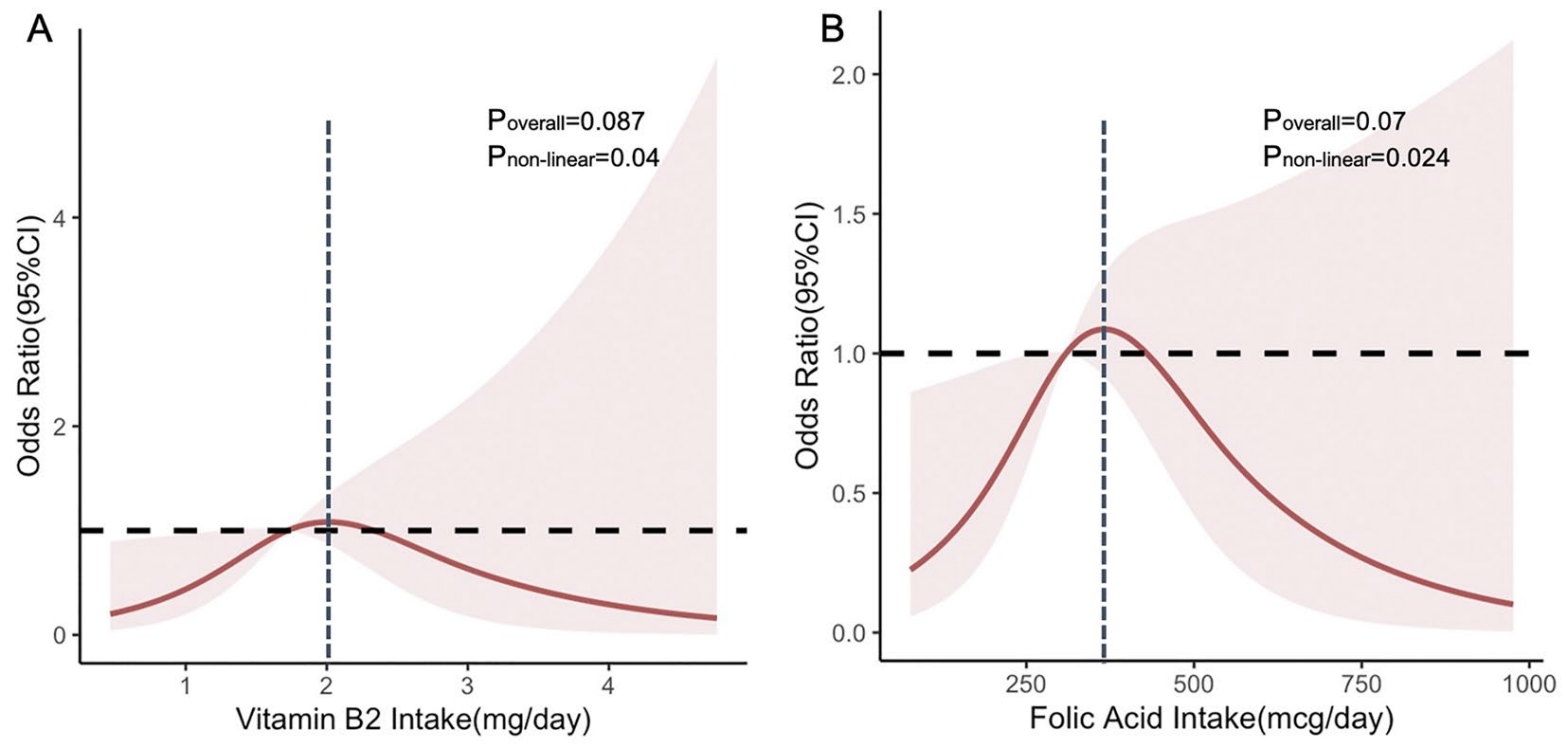
Restricted cubic spline regression of the association between B vitamin intake and the odds ratio of glaucoma based on ISGEO criteria in females after controlling for age, race, educational level, smoking, diabetes, cataract surgery, daily total energy, caffeine intake and interacted vitamin b. **A** Vitamin B1; **B** Folic acid. *ISGEO* International Society Geographical and Epidemiological Ophthalmology.

Restricted cubic spline regression revealed a nonlinear association (Fig. 2) between vitamin B2, B9 intake and glaucoma diagnosed by ISGEO criteria in females after controlling for all confounders. Within the higher range of vitamin B2 and folic acid intake, there was a slight decrease in glaucoma prevalence. The highest risk was reached at approximately 2.021 mg/day (vitamin B2) and 366.133 mcg/day (folic acid), and then gradually decreased (vitamin B2: P for nonlinearity = 0.040; folic acid: P for nonlinearity = 0.024). However, no nonlinear relationship was observed between the intake of other B vitamins and glaucoma (Supplement Fig. S1–S6).

## Discussion

This was a study on glaucoma based on self-report and ISGEO criteria associated with the dietary intake of B vitamins in a population of Americans aged over 40 years. In logistic regression analysis, reference values of quartiles were determined based on RDAs. This analysis allowed us to compare the risk of glaucoma in people who consume different levels of B vitamins and that in people who consume the RDAs. The overall analysis revealed that daily consumption of vitamin B1 was related to glaucoma prevalence as diagnosed by self-report. Moreover, there was a significant P trend for vitamin B12 intake. After sex-stratified analysis, the association between vitamin B1 and self-reported glaucoma persisted in males, and we also found that high vitamin B2 intake was associated with a reduced risk of self-reported glaucoma. Further research found that the daily intake of niacin was associated with glaucoma prevalence according to the overall analysis based on ISGEO criteria. Furthermore, higher intake of vitamin B6 was associated with a reduced risk of glaucoma based on ISGEO criteria in males. Restricted cubic spline regression revealed a nonlinear association between vitamin B2, folic acid intake, and glaucoma based on ISGEO criteria in females after controlling for all variables.

Vitamins B1 and B2 can function as coenzymes in amino acid metabolism, cell division and growth, DNA synthesis, and repair in human cells^10^. In addition, vitamin B1 is involved in glucose metabolism and neurotransmitter synthesis, and has an antioxidant effect on nerve cells^27^. Sulbutiamine, a synthetic derivative of vitamin B1, exerts protective effects on RGCs^28^. Vitamin B2 is effective in migraine treatment, which is often a predisposing factor for glaucoma attack^10,28^. Our study found that when vitamin B1 intake was in the third (1.386–1.855 mg/day) quartile, compared to that in the second quartile, which is the range of RDAs, the odds of self-reported glaucoma decreased. When all the adjustments were made, the connection did not disappear. The Rotterdam study reported that medium and high doses of vitamin B1 had the strongest protective effect on POAG, which supported our conclusion^29^. According to a Korean study on the association between nutrient intake and POAG, a low intake of vitamins B1 and B2 may be associated with an increased likelihood of glaucoma^30^. In our study, the association between the intake of vitamins B1, B2 and self-reported glaucoma appeared to be stronger in males. In females, vitamin B2 intake was nonlinearly associated with ISGEO-diagnosed glaucoma, and glaucoma risk decreased with higher intake.

Several studies have reported an association between serum homocysteine levels and normal-tension glaucoma, POAG, and PEXG^31,32^. Folic acid and vitamins B6 and B12 can help improve homocysteine metabolism, thereby reducing vascular endothelial function injury, ganglion cell apoptosis, extracellular matrix alterations, lysyl oxidase activity, and oxidative stress^33^. Our findings showed a significant P trend for vitamin B12 intake in self-reported glaucoma analysis. This result implied that the risk of self-reported glaucoma gradually decreases with vitamin B12 intake from Q1 to Q4. Our findings suggested that in males, higher vitamin B6 intake has a negative relationship with the odds of glaucoma based on ISGEO criteria at the third quartile (1.914–2.669 mg/day) compared to that in the second quartile (the range where the RDAs are located). In addition, Giaconi et al.^34^ and Coleman et al.^35^ did not observe a correlation between folic acid intake and POAG in older women, which was similar to our conclusion from the logistic regression analysis. However, in further analysis of females, we found a non-linear relationship between folate intake and glaucoma based on ISGEO criteria.

Niacin, vitamin B3, has been confirmed to have neuroprotective effects in cell and animal experiments, can increase mitochondrial size and dynamics and provide effectiveness for neuroprotective therapy for glaucoma^36^. A study based on NHANES in South Korea, which analyzed 24-h recalled dietary data and identified glaucoma by ophthalmic examination, found that high levels of dietary niacin were associated with a reduced risk of glaucoma in people over 40 years of age. The results of Taechameekietichai et al.^12^ and Lee et al.^13^ support the association between niacin intake and glaucoma. Our findings found that every 1 mg increase in dietary niacin intake was associated with a 6 percent reduction in the risk of glaucoma diagnosed by ISGEO criteria. However, after sex- stratification analysis, the relationship was not significant.

This study conducted a sex-stratified analysis of the association between vitamin B and glaucoma and found differences between the two sexes. We observed an association between higher intakes of vitamin B1, B2 and the risk of glaucoma on self-reported criteria in males. In glaucoma diagnosed based on ISGEO criteria, dietary vitamin B6 intake was associated with the risk of glaucoma in males, while vitamin B2 and folic acid were non-linearly associated with the risk of glaucoma in females. This suggested that sex hormones play a role in metabolism of B vitamins and might influence glaucoma risk^37^. Our study could not prove a sex-specific effect of B vitamins on glaucoma; hence, more research would be needed to explore the mechanisms behind phenomenon.

The strength of our study design is the reliable data sample obtained from the US population between 2005 and 2008, which could be adjusted for potential confounding factors, rendering it representative and persuasive. Additionally, our reference values were used as RDAs proposed by the National Institutes of Health, which had certain clinical significance. However, our study also had some limitations. NHANES is a cross-sectional study, although, we did not observe valid evidence of a causal relationship between B vitamins and glaucoma prevalence. Moreover, the possibility of dietary changes owing to glaucoma onset cannot be ruled out. The main outcome variable in this study was self-reported glaucoma, and glaucoma diagnosis was based solely on participant questionnaire responses. This method lacked rigorous ophthalmic examinations and could have resulted in information bias during data analysis. However, some studies have reported high consistency between self-reported glaucoma and participant medical records in glaucoma diagnosis^38,39^. Furthermore, our secondary outcome variable was glaucoma based on categories 1 and 2 of the ISGEO criteria. The ISGEO standard included fundus vertical CDR assessment and FDT examinations, which partially reduced information bias and misclassification bias incidence, despite the absence of IOP values. Optic neuropathy predates visual field changes in fundus imaging in glaucoma^40^. Therefore, the ISGEO criteria may not apply to patients with mild glaucoma. In addition, the prevalence of self-reported glaucoma was 6.59%, and that of ISGEO standard diagnosis was 3.31%. The former was likely to include a significant proportion of false positive cases, the latter might be closer to the current prevalence of glaucoma^1,2,41^. Daily intake levels of B vitamins calculated based on nutrient sources from two 24-h dietary interviews, may have been influenced by recall and information bias. In addition, bioavailability varies among individuals. Therefore, serum vitamin levels should be investigated, and prospective cohort studies should be designed to provide more objective evidence.

Based on this large cross-sectional study, we concluded that on the self-reported criteria, vitamin B1 and B12 intake was associated with the odds of glaucoma; while in males, higher intake of vitamin B1 and B2 had negative relationship with glaucoma risk. On the ISGEO criteria, the risk of glaucoma decreased with the increase in niacin intake; while in males, there was a significant association between vitamin B6 intake and glaucoma; in females, dietary intake of vitamin B2 and folic acid had obviously nonlinear relationship with the odds of glaucoma.

### Supplementary Information

Supplementary Information 1.

Supplementary Information 2.

Supplementary Information 3.

Supplementary Information 4.

Supplementary Information 5.

Supplementary Information 6.

Supplementary Information 7.

Supplementary Information 8.

Supplementary Information 9.

Supplementary Information 10.

## Data Availability

Data can be available from the database website https://www.cdc.gov/nchs/nhanes.
