# Supplementary material for: Association of dietary intake of B vitamins with glaucoma
Source: Sci Rep. 2024 Apr 12;14:8539. doi: 10.1038/s41598-024-58526-5 (PMC11014949; doi:10.1038/s41598-024-58526-5)
Supplement: Supplementary file 7 — Supplementary Information 7. [file 41598_2024_58526_MOESM7_ESM.pdf]

Association of dietary intake of B vitamins with glaucoma

Author names

Jingjing Hou[1,2], Yu Wen[1,2], Sijia Gao[1,2], Zhengxuan Jiang[1], Liming Tao[1]*

Affiliations

[1]Department of Ophthalmology, The Second Affiliated Hospital of Anhui Medical University, 678 Furong Road, Hefei, Anhui, China

[2]Department of clinical medicine, The Second School of Clinical Medicine, Anhui Medical University, 81 Meishan Road, Hefei, Anhui, China

Corresponding author

Liming Tao, MD

Department of Ophthalmology, The Second Affiliated Hospital of Anhui Medical University, 678 Furong Road, Hefei, Anhui, China

taoliming@ahmu.edu.cn

ORCiD

0000-0002-0942-5246

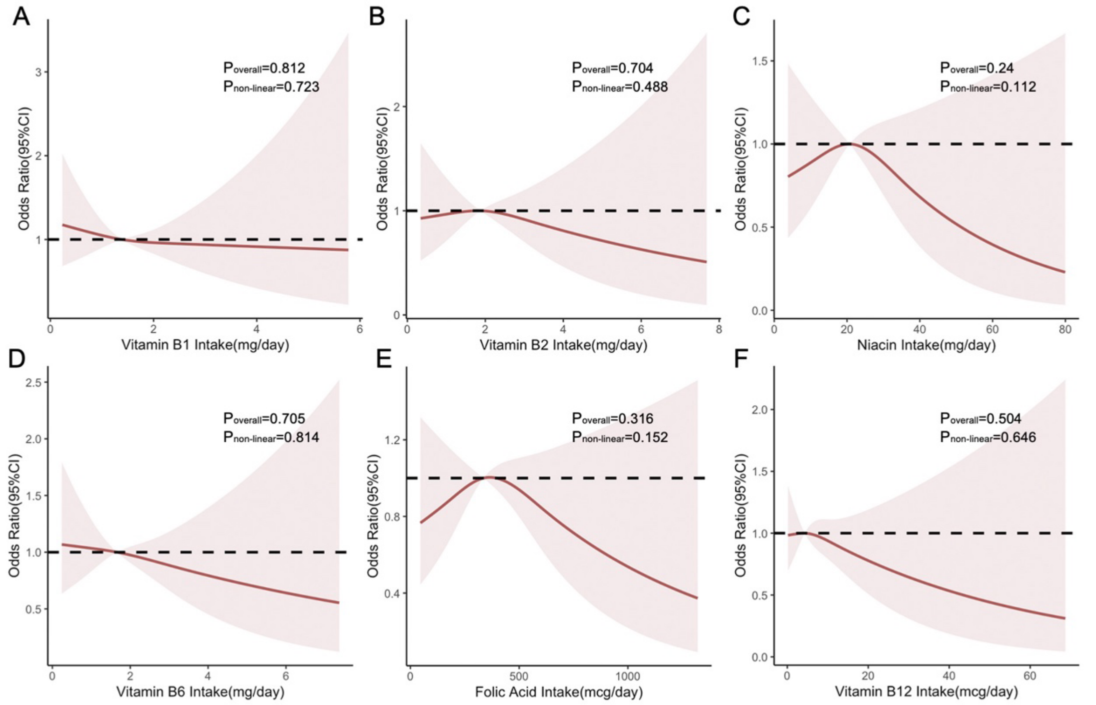

Supplement Fig S1 Restricted cubic spline regression of the association between B vitamin intake and the odds ratio of self-glaucoma after controlling for age, sex, race, educational level, smoking, diabetes, cataract surgery, daily total energy, caffeine intake and interacted vitamin b. (A) Vitamin B1; (B) Vitamin B2; (C) Niacin; (D) Vitamin B6; (E) Folic acid; (F) Vitamin B12

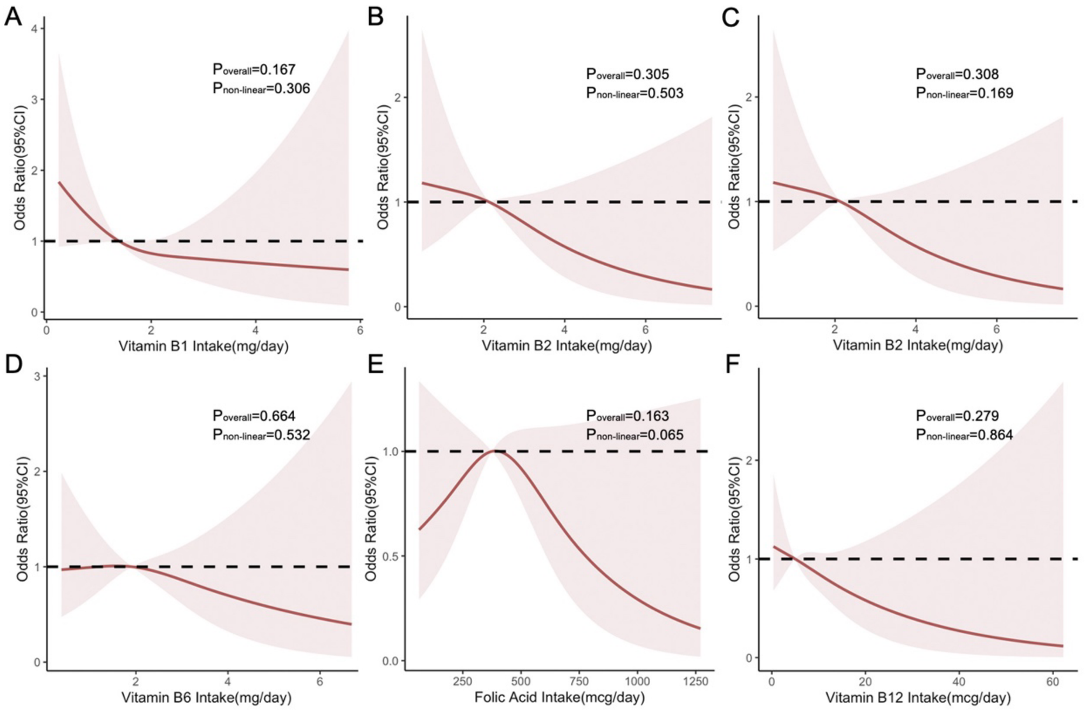

Supplement Fig S2 Restricted cubic spline regression of the association between B vitamin intake and the odds ratio of self-glaucoma in males after controlling for age, race, educational level, smoking, diabetes, cataract surgery, daily total energy, caffeine intake and interacted vitamin b. (A) Vitamin B1; (B) Vitamin B2; (C) Niacin; (D) Vitamin B6; (E) Folic acid; (F) Vitamin B12

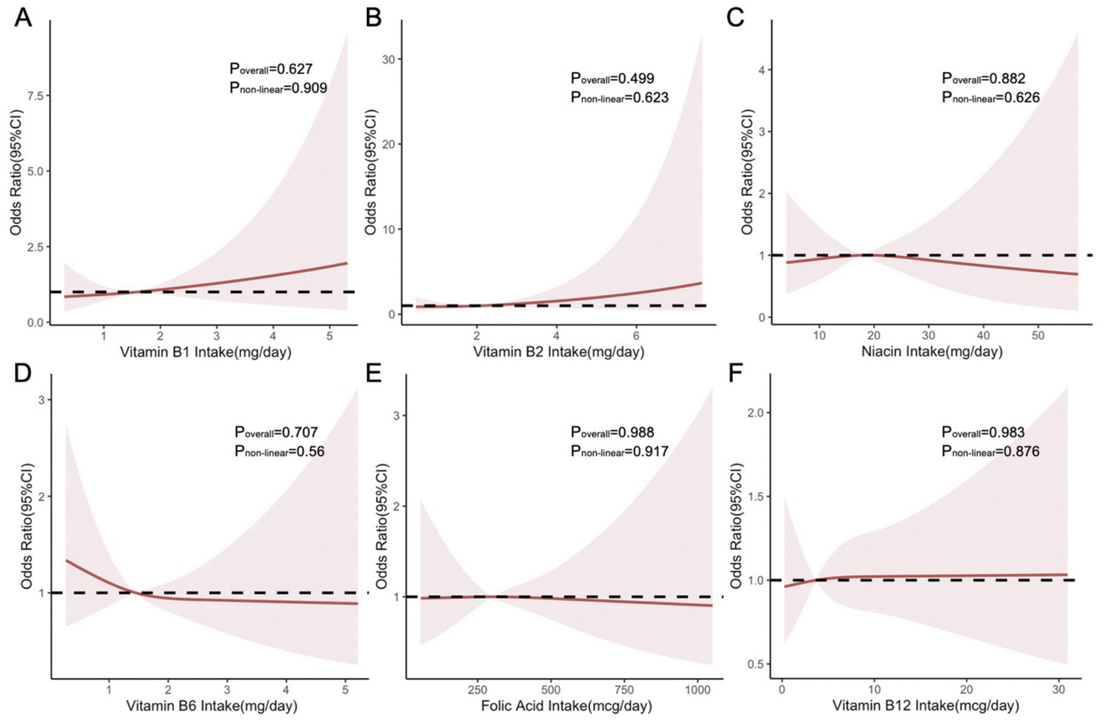

Supplement Fig S3 Restricted cubic spline regression of the association between B vitamin intake and the odds ratio of self-glaucoma in females after controlling for age, race, educational level, smoking, diabetes, cataract surgery, daily total energy, caffeine intake and interacted vitamin b. (A) Vitamin B1; (B) Vitamin B2; (C) Niacin; (D) Vitamin B6; (E) Folic acid; (F) Vitamin B12

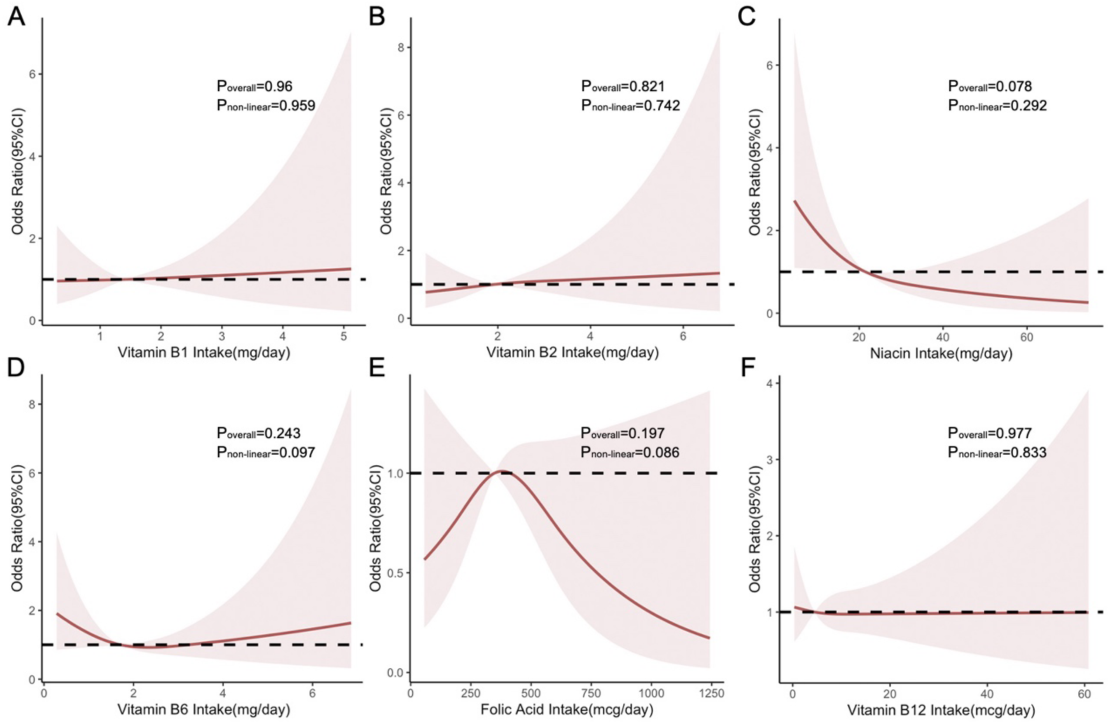

Supplement Fig S4 Restricted cubic spline regression of the association between B vitamin intake and the odds ratio of glaucoma based on ISGEO criteria after controlling for age, sex, race, educational level, smoking, diabetes, cataract surgery, daily total energy, caffeine intake and interacted vitamin b. (A) Vitamin B1; (B) Vitamin B2; (C) Niacin; (D) Vitamin B6; (E) Folic acid; (F) Vitamin B12

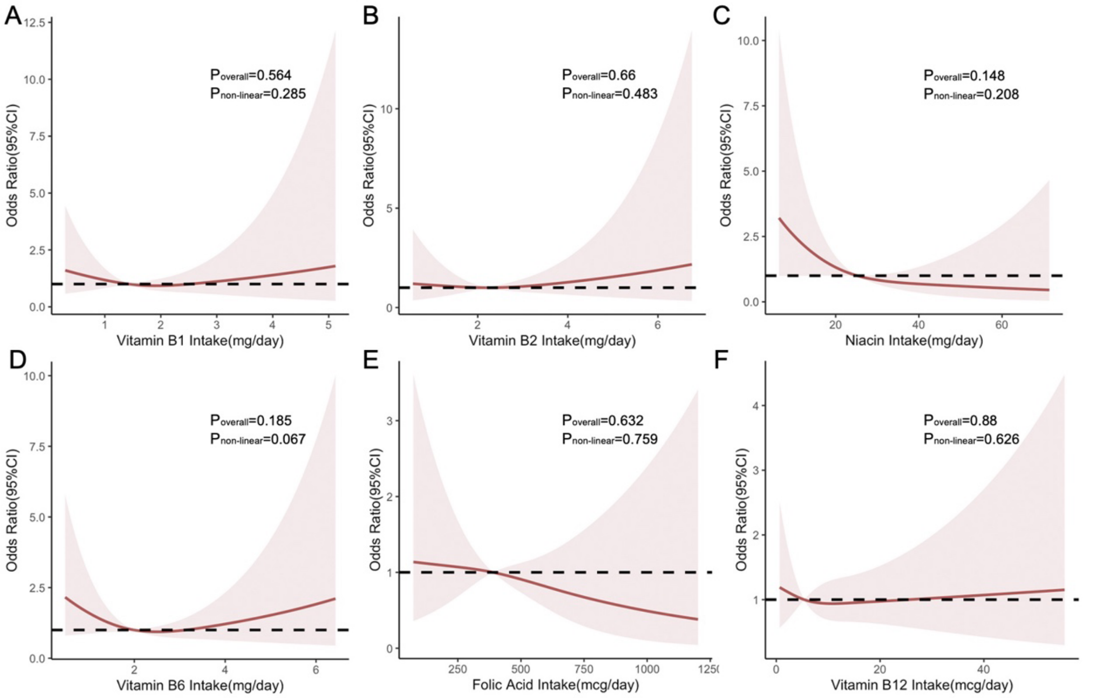

Supplement Fig S5 Restricted cubic spline regression of the association between B vitamin intake and the odds ratio of glaucoma based on ISGEO criteria in males after controlling for age, race, educational level, smoking, diabetes, cataract surgery, daily total energy, caffeine intake and interacted vitamin b. (A) Vitamin B1; (B) Vitamin B2; (C) Niacin; (D) Vitamin B6; (E) Folic acid; (F) Vitamin B12

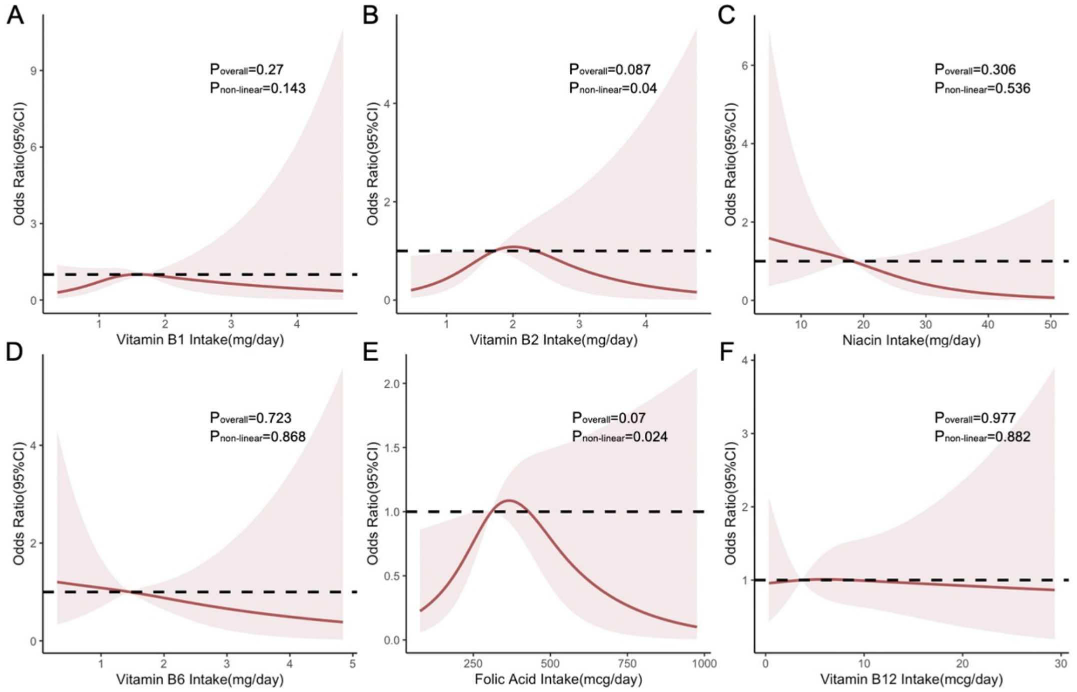

Supplement Fig S6 Restricted cubic spline regression of the association between B vitamin intake and the odds ratio of glaucoma based on ISGEO criteria in females after controlling for age, race, educational level, smoking, diabetes, cataract surgery, daily total energy, caffeine intake and interacted vitamin b. (A) Vitamin B1; (B) Vitamin B2; (C) Niacin; (D) Vitamin B6; (E) Folic acid; (F) Vitamin B12
