# Supplementary material for: Association of dietary intake of B vitamins with glaucoma
Source: Sci Rep. 2024 Apr 12;14:8539. doi: 10.1038/s41598-024-58526-5 (PMC11014949; doi:10.1038/s41598-024-58526-5)
Supplement: Supplementary file 8 — Supplementary Information 8. [file 41598_2024_58526_MOESM8_ESM.docx]

**Table S1** Recommended daily allowances (RDAs) for people aged 40 or greater proposed by the National Institutes of Health

| Nutrient | Male | Female | Overall |
| --- | --- | --- | --- |
| Vitamin B1(mg/day) | 1.2 | 1.1 | 1.15 |
| Vitamin B2(mg/day) | 1.3 | 1.1 | 1.2 |
| Niacin(mg/day) | 16 | 14 | 15 |
| Vitamin B6(mg/day) | 1.3-1.7 | 1.3-1.5 | 1.3-1.7 |
| Folic acid(mcg/day) | 400 | 400 | 400 |
| Vitamin B12(mcg/day) | 2.4 | 2.4 | 2.4 |
