# Supplementary material for: Association of dietary intake of B vitamins with glaucoma
Source: Sci Rep. 2024 Apr 12;14:8539. doi: 10.1038/s41598-024-58526-5 (PMC11014949; doi:10.1038/s41598-024-58526-5)
Supplement: Supplementary file 9 — Supplementary Information 9. [file 41598_2024_58526_MOESM9_ESM.docx]

**Table S2** Association between daily dietary intake of B vitamins and self-reported glaucoma prevalence in females

|  | Crude Model  OR (95% CI) | P Value | Model I  OR (95% CI) | P Value | Model II  OR (95% CI) | P Value |
| --- | --- | --- | --- | --- | --- | --- |
| Vitamin B1 |  |  |  |  |  |  |
| Continuous | 0.90(0.66,1.23) | 0.51 | 1.03(0.74,1.43) | 0.877 | 1.04(0.70,1.55) | 0.853 |
| Q1 | 0.63(0.36,1.10) | 0.103 | 0.62(0.34,1.12) | 0.112 | 0.63(0.33,1.20) | 0.159 |
| Q2 | ref |  | ref |  | ref |  |
| Q3 | 0.72(0.42,1.23) | 0.227 | 0.72(0.41,1.28) | 0.266 | 0.76(0.42,1.37) | 0.358 |
| Q4 | 0.70(0.41,1.19) | 0.185 | 0.84(0.47,1.48) | 0.543 | 0.93(0.50,1.74) | 0.829 |
| p trend |  | 0.864 |  | 0.80 |  | 0.733 |
| Vitamin B2 |  |  |  |  |  |  |
| Continuous | 0.86(0.69,1.08) | 0.202 | 1.03(0.82,1.30) | 0.773 | 1.10(0.80,1.52) | 0.555 |
| Q1 | ref |  | ref |  | ref |  |
| Q2 | 0.79(0.46,1.36) | 0.392 | 0.92(0.52,1.60) | 0.758 | 0.99(0.54,1.79) | 0.965 |
| Q3 | 0.96(0.57,1.61) | 0.866 | 1.16(0.67,1.99) | 0.603 | 1.29(0.68,2.42) | 0.433 |
| Q4 | 0.82(0.49,1.37) | 0.452 | 1.20(0.71,2.00) | 0.513 | 1.55(0.78,3.06) | 0.209 |
| p trend |  | 0.576 |  | 0.594 |  | 0.172 |
| Niacin |  |  |  |  |  |  |
| Continuous | 0.98(0.96,1.00) | 0.076 | 1.00(0.98,1.02) | 0.849 | 1.01(0.98,1.04) | 0.655 |
| Q1 | 1.50(0.86,2.64) | 0.156 | 1.45(0.80,2.63) | 0.216 | 1.37(0.72,2.63) | 0.336 |
| Q2 | ref |  | ref |  | ref |  |
| Q3 | 1.40(0.79,2.49) | 0.252 | 1.77(0.95,3.28) | 0.071 | 1.81(0.96,3.39) | 0.066 |
| Q4 | 0.91(0.49,1.68) | 0.761 | 1.37(0.70,2.68) | 0.362 | 1.52(0.70,3.30) | 0.29 |
| p trend |  | 0.084 |  | 0.97 |  | 0.509 |
| Vitamin B6 |  |  |  |  |  |  |
| Continuous | 0.92(0.73,1.17) | 0.518 | 0.97(0.76,1.22) | 0.782 | 0.92(0.69,1.24) | 0.594 |
| Q1 | 0.92(0.54,1.57) | 0.771 | 0.89(0.51,1.55) | 0.675 | 0.96(0.54,1.70) | 0.882 |
| Q2 | ref |  | ref |  | ref |  |
| Q3 | 0.72(0.41,1.26) | 0.251 | 0.80(0.45,1.43) | 0.449 | 0.80(0.44,1.45) | 0.465 |
| Q4 | 0.78(0.46,1.32) | 0.356 | 0.82(0.48,1.42) | 0.485 | 0.79(0.42,1.50) | 0.471 |
| p trend |  | 0.431 |  | 0.482 |  | 0.596 |
| Folic Acid |  |  |  |  |  |  |
| Continuous | 1.00(1.00,1.00) | 0.132 | 1.00(1.00,1.00) | 0.351 | 1.00(1.00,1.00) | 0.429 |
| Q1 | 1.18(0.66,2.08) | 0.577 | 1.08(0.60,1.93) | 0.792 | 1.17(0.64,2.13) | 0.615 |
| Q2 | 1.57(0.89,2.76) | 0.118 | 1.38(0.76,2.50) | 0.286 | 1.41(0.78,2.54) | 0.259 |
| Q3 | ref |  | ref |  | ref |  |
| Q4 | 0.78(0.44,1.38) | 0.387 | 0.81(0.45,1.45) | 0.471 | 0.81(0.45,1.47) | 0.489 |
| p trend |  | 0.008 |  | 0.06 |  | 0.146 |
| Vitamin B12 |  |  |  |  |  |  |
| Continuous | 0.99(0.96,1.03) | 0.616 | 0.99(0.95,1.03) | 0.516 | 1.00(0.96,1.03) | 0.809 |
| Q1 | 1.61(0.88,2.94) | 0.126 | 1.45(0.77,2.72) | 0.251 | 1.48(0.77,2.84) | 0.239 |
| Q2 | ref |  | ref |  | ref |  |
| Q3 | 1.44(0.78,2.68) | 0.247 | 1.38(0.72,2.64) | 0.329 | 1.46(0.76,2.80) | 0.256 |
| Q4 | 1.36(0.75,2.47) | 0.306 | 1.21(0.66,2.25) | 0.536 | 1.41(0.73,2.72) | 0.30 |
| p trend |  | 0.929 |  | 0.793 |  | 0.744 |

Model I adjusted for age, race and educational level

Model II adjusted for age, race, educational level, smoking, diabetes, cataract surgery, daily total energy, caffeine intake and interacted vitamin b
