# Supplementary material for: Association of dietary intake of B vitamins with glaucoma
Source: Sci Rep. 2024 Apr 12;14:8539. doi: 10.1038/s41598-024-58526-5 (PMC11014949; doi:10.1038/s41598-024-58526-5)
Supplement: Supplementary file 10 — Supplementary Information 10. [file 41598_2024_58526_MOESM10_ESM.docx]

**Table S3** Association between daily B vitamins and glaucoma diagnosed by ISGEO criteria in females

|  | Crude Model  OR (95% CI) | P Value | Model I  OR (95% CI) | P Value | Model II  OR (95% CI) | P Value |
| --- | --- | --- | --- | --- | --- | --- |
| Vitamin B1 |  |  |  |  |  |  |
| Continuous | 0.98(0.48,2.02) | 0.96 | 1.07(0.53,2.18) | 0.846 | 1.33(0.72,2.46) | 0.359 |
| Q1 | 0.57(0.17,1.93) | 0.362 | 0.57(0.16,2.04) | 0.388 | 0.38(0.10,1.48) | 0.161 |
| Q2 | ref |  | ref |  | ref |  |
| Q3 | 1.46(0.56,3.81) | 0.44 | 1.48(0.57,3.83) | 0.414 | 1.70(0.61,4.73) | 0.307 |
| Q4 | 0.55(0.17,1.76) | 0.316 | 0.61(0.18,2.03) | 0.424 | 0.97(0.23,4.09) | 0.965 |
| p trend |  | 0.87 |  | 0.993 |  | 0.184 |
| Vitamin B2 |  |  |  |  |  |  |
| Continuous | 0.62(0.40,0.95) | 0.027 | 0.68(0.43,1.07) | 0.098 | 0.77(0.42,1.41) | 0.399 |
| Q1 | ref |  | ref |  | ref |  |
| Q2 | 0.79(0.26,2.35) | 0.668 | 0.81(0.24,2.73) | 0.739 | 1.05(0.30,3.64) | 0.941 |
| Q3 | 0.70(0.26,1.75) | 0.414 | 0.75(0.26,2.16) | 0.591 | 1.06(0.27,4.18) | 0.937 |
| Q4 | 0.55(0.19,1.60) | 0.269 | 0.68(0.22,2.04) | 0.49 | 1.48(0.26,8.41) | 0.656 |
| p trend |  | 0.254 |  | 0.36 |  | 0.593 |
| Niacin |  |  |  |  |  |  |
| Continuous | 0.95(0.91,1.00) | 0.054 | 0.96(0.92,1.01) | 0.118 | 0.94(0.87,1.02) | 0.138 |
| Q1 | 0.64(0.24,1.69) | 0.365 | 0.65(0.24,1.77) | 0.402 | 0.68(0.23,1.96) | 0.471 |
| Q2 | ref |  | ref |  | ref |  |
| Q3 | 0.53(0.20,1.41) | 0.201 | 0.58(0.22,1.55) | 0.28 | 0.57(0.20,1.62) | 0.293 |
| Q4 | 0.28(0.08,0.94) | 0.04 | 0.35(0.10,1.17) | 0.087 | 0.34(0.07,1.73) | 0.196 |
| p trend |  | 0.083 |  | 0.143 |  | 0.442 |
| Vitamin B6 |  |  |  |  |  |  |
| Continuous | 0.87(0.56,1.35) | 0.524 | 0.89(0.56,1.41) | 0.618 | 1.08(0.62,1.89) | 0.782 |
| Q1 | 0.80(0.29,2.19) | 0.658 | 0.79(0.28,2.24) | 0.654 | 0.74(0.24,2.31) | 0.61 |
| Q2 | ref |  | ref |  | ref |  |
| Q3 | 0.81(0.28,2.30) | 0.689 | 0.85(0.28,2.40) | 0.76 | 0.97(0.34,2.72) | 0.947 |
| Q4 | 0.71(0.23,2.15) | 0.543 | 0.74(0.30,2.26) | 0.591 | 1.05(0.30,3.69) | 0.936 |
| p trend |  | 0.727 |  | 0.726 |  | 0.533 |
| Folic Acid |  |  |  |  |  |  |
| Continuous | 1.00(1.00,1.00) | 0.665 | 1.00(1.00,1.00) | 0.781 | 1.00(1.00,1.00) | 0.963 |
| Q1 | 0.64(0.21,1.93) | 0.424 | 0.62(0.19,1.99) | 0.418 | 0.44(0.11,1.73) | 0.241 |
| Q2 | 1.05(0.38,2.87) | 0.928 | 0.92(0.31,2.70) | 0.873 | 0.89(0.29,2.73) | 0.833 |
| Q3 | ref |  | ref |  | ref |  |
| Q4 | 0.76(0.27,2.11) | 0.595 | 0.76(0.26,2.17) | 0.605 | 0.81(0.26,2.53) | 0.714 |
| p trend |  | 0.947 |  | 0.935 |  | 0.414 |
| Vitamin B12 |  |  |  |  |  |  |
| Continuous | 1.01(0.99,1.04) | 0.316 | 1.02(0.99,1.05) | 0.186 | 1.02(0.99,1.06) | 0.212 |
| Q1 | 1.37(0.49,3.81) | 0.55 | 1.31(0.47,3.69) | 0.605 | 1.34(0.48,3.75) | 0.576 |
| Q2 | ref |  | ref |  | ref |  |
| Q3 | 1.79(0.58,5.55) | 0.31 | 1.90(0.62,5.78) | 0.258 | 2.39(0.72,7.93) | 0.153 |
| Q4 | 1.81(0.61,5.40) | 0.288 | 1.97(0.70,5.51) | 0.197 | 2.85(0.83,9.74) | 0.095 |
| p trend |  | 0.182 |  | 0.156 |  | 0.058 |

Model I adjusted for age, race and educational level

Model II adjusted for age, race, educational level, smoking, diabetes, cataract surgery, daily total energy, caffeine intake and interacted vitamin b
